# Editorial: Values shared by journals of learned societies, associations and scientific institutions in animal science^*^

**DOI:** 10.1093/jas/skae379

**Published:** 2025-03-03

**Authors:** I Ortigues-Marty, I Louveau, G Bee, J W Oltjen, P J Kononoff, J A A McArt, C Thomas, B D Fairchild, M Kogut, E Huff-Lonergan

**Affiliations:** UMR1213 Herbivores, INRAE, 63122 Saint-Genès-Champanelle, France; PEGASE, INRAE, Institut Agro, 35590, Saint Gilles, France; Agroscope Posieux, Switzerland; Department of Animal Science, University of California, Davis, CA, USA; Department of Animal Science, University of Nebraska-Lincoln, Lincoln, NE, USA; Department of Population Medicine & Diagnostic Sciences, Cornell University, Ithaca, NY, USA; European Federation of Animal Science, Via G. Tomassi 3, 00161 Roma, Italy; Department of Poultry Science Department, University of Georgia, Athens, GA, USA; USDA-ARS Southern Plains Agricultural Research Center, 2881 F&B Road, College Station TX, USA; Department of Animal Science, 914 Stange Road, Iowa State University, Ames, IA, USA

Scientific publishing has undergone a tremendous change in recent years. We, a group of Editors-in-Chief of scientific journals owned by scientific bodies, want to communicate some of our values. We represent animal, animal – open science, animal – science proceedings, JDS Communications, Journal of Animal Science, Journal of Applied Poultry Research, Journal of Dairy Science, Poultry Science and Translational Animal Science. Our values motivate our involvement in society-, association-or scientific institution-owned journals in animal science and shape our practices in scientific publishing, in the light of the tremendous changes in the land-scape of scientific publishing over the last decade.

## A changing landscape

In the past, most scientific journals were typically owned and managed by learned societies, associations and research organisations. Publication costs were mostly borne by subscriptions and paid by libraries. This system, however, has limitations. For instance, under a subscription-based model, published articles are not freely available, which limits accessibility to scientific works and the development of science throughout the world. Sometimes, sound but non-original research would not be published because of the stringent peer-review process, which contributed to the reproducibility crisis (Baker, 2016) by not publishing studies that tested the reproducibility of published results.

The move towards Open Science and the rapid development of Open Access publishing has drastically changed the landscape of scientific publication over the last 10 years. An array of new journals has been launched by an increasing diversity of owners, and new publishing models encouraged new practices. Under the Open Access model, the publication cost is borne by the authors. A major advantage of this model is that published articles are freely available to anyone. A serious challenge to authors is the need to secure funds to publish. This challenge is especially hard for authors with few financial resources and/or from countries or institutions with lower income. In the traditional subscription-based model, readers were the clients. Publishers (journals) thus had to seek readers to meet their expectations (supply them with good quality articles), which allowed the income to follow the product. With authors becoming the client, the income comes before the product, and as a result, scientific publishing is moving towards an author-driven market, less risky to the publishers and owners. Rejecting an article implies a loss of income. Researchers, i.e. authors, are also under pressure to publish to advance their career. They desire their papers to be published, and published fast, a practice that may come at the expense of an in-depth and thorough peer-review process.

Another challenge that journals face is the availability of reviewers that contributes to limiting scientific debate. The peer-review system is perceived as conferring a ‘‘mark of quality” and relies on the collective expertise of the scientific community (van Milgen et al., 2023). However, a system-wide challenge that virtually all journals face is that many scientists have limited time to act as reviewers for papers of other scientists, which stifles the peer-review process and often results in frustration for authors. In parallel, the number of publications per year in animal and veterinary science doubled between 2016 and 2021, amounting to 12,000 per year in 2021, implying that the number of reviewers should have quadrupled. The practice of posting preprints (non-peer-reviewed manuscripts) in repositories, sometimes prior to submission to a peer-reviewed journal, is also developing. Preprints expedite the sharing of research results within the scientific community and larger society, and aid authors in establishing intellectual property. However, these publications do not undergo a rigorous peer review, hence, a greater responsibility is put on readers to make their own analysis, sometimes through dedicated discussion platforms. Many readers are not fully aware of these discussions and devote less and less time to critical reading of the scientific literature. The combination of insufficient reviewers and limited scientific debate on the contents of preprints threatens to weaken the soundness of published research. It can endanger a crucial driver of scientific progress that is the critical process of open scientific debate. The healthy debate of research rationales, questions/hypotheses, results and interpretations allows constructive challenge based on scientific evidence. Diminished scientific debate has the potential to contribute to the growing mistrust of society towards science.

## Our values

Society- or scientific institution-owned journals have a role to play in this changing landscape. One of the key roles of scientific societies and institutions is to foster, promote and defend scientific integrity within its community of scientists. The values that we defend constitute a direction for our actions. Each journal implements them at its own pace and its own way.

### Promote and defend scientific integrity

As representatives of societies or institutions, our journals adhere to the international standards for responsible research publication (Wager and Kleinert, 2011) and stress the values that are the foundation of our publication strategies and practices. As society, association or scientific institution-owned journals, science and responsible research drive our activities, not financial return.

### For scientists, by scientists

Our motto is ‘For scientists, by scientists’. The publication of sound and reliable research is our foremost objective. Peer review is a vital element to evaluate the reliability of research, and we take it very seriously. However, solid peer review takes time, and we value a thorough, fair and transparent peer-review process over less than rigorous peer review and rapid time to decision and publication. We invite scientists who publish in our journals to also review and thus return the effort and time of other scientists in the community. By accepting an invitation to review research of their colleagues, scientists ensure well-grounded research is published. We recognise that even after peer review, a published article can and should be challenged by new evidence, and some of our societies and institutions are keen to develop online discussion platforms to allow healthy discussion of science.

The cost of running journals is borne by learned societies, associations or scientific institutions, and their publishers, and financial return is necessary for journals to operate. We support transparency on the use of journal income by journal owners. Societies, associations and institutions re-invest proceeds to cover costs borne by the editorial activity, and into activities that promote the science of the authors they publish and of their members through the organisation of workshops and conferences, educational opportunities, grants, research projects, etc.

Many factors are important in evaluating journals. We recognise that the impact factor is an index of the reputation of a journal but not necessarily an index of the quality of individually published articles. Some scientific institutions, which own our journals, signed the Dora declaration (https://sfdora.org/) and consider that researchers should no longer be evaluated on the impact factor of journals they choose to publish in. We are progressively engaging in Open Science and developing new practices dedicated to ensuring the continued publication of sound science.

### Scientific integrity in research and publication

Scientific integrity in research and publication is a prerequisite for author and reader trust. Article contents include declarations made by authors on why and how they conducted their research, about authors’ and funders’ contribution, conflicts of interest, access to data etc. Declarations are not factual proofs, and trust and openness are necessary between authors, editors, and reviewers. We strive for the highest ethical standards, requesting research using animals or humans to be done in compliance with national or international legislation, requiring all journal actors to comply with a strict code of duty and obligation, applying ethical quality controls on the submissions, and expecting honesty and transparency from all authors.

### Reproducibility of published research

The need to ensure reproducibility of research (Baker, 2016) touches all research fields, including animal science, and we, as scientific journal editors, defend reproducibility in published research. We believe that each research result should include detailed reporting of the material and methods, fair and equitable access to data and codes, and our journals are making steps in this direction. Most learned societies and scientific institutions have journals that support the publication of methods, data, or short communication papers. We encourage the deposition of data in official repositories, applying the FAIR principles, leaving the responsibility to authors to select the repository of their choice. Although the originality of research results, otherwise named ‘significant addition to existing scientific knowledge’, contributes to advancing the frontlines of knowledge, testing whether a research result is reproducible is critical for its potential applications and for future research. Societies or scientific institution-owned journals offer publication of confirmatory research as well as research that provides conclusions of ‘negative’ results, which are essential to advancing scientific knowledge.

### Service to scientists

Providing services to researchers is also important to societies, scientific institutions and their journals. Learned societies and scientific institutions, as well as their scientific journals, have a role to play in helping authors develop their understanding of scientific practice and effectively sharing their results. They offer scientists a range of activities (e.g. conferences and workshops) and thereby opportunities to actively develop their network and to debate and discuss research with local and international colleagues. Journals from learned societies, associations and scientific institutions are important to institutions that value service to the scientific community and to the society as a whole and use publishing activities in their evaluation of professional accomplishments. They provide guides of good practice to authors, and journal staff strive to be as helpful as possible to authors and reviewers. This is accompanied by balanced and professional communication regarding our journals and published works.

## The future

The future of society and scientific institution journals depends on scientists. Our journals are managed by active researchers who devote part of their professional time to scientific publishing. Many are doing it voluntarily while a few receive an honorarium. Being researchers, authors, reviewers and sometimes editors themselves, they are in close touch with scientific life and aware of the continuous evolution of publishing. They are under pressure to meet all criteria set out by their employers and expectations from authors and reviewers, but they are firmly convinced of their engagement in scientific publishing for learned societies, associations or scientific institutions.

To conclude, we are very optimistic about the future of scientific publishing and the role our scientific societies or institutions play in advancing knowledge, innovating, and supporting the careers of scientists. Journals associated with societies or scientific institutions recognise the critical part they play in the development of new understandings and technologies not only through publishing research but also by supporting the activities of scientists active in the field. Our societies/institutions and their journals, by adhering to our values, contribute significantly to ensuring the long-term sustainability of animal sciences and of the people who work in the scientific field.
